# Some Aspects of Medical Science

**Published:** 1894-07-07

**Authors:** 


### Some Aspects of Medical Science.

In our last paper we discussed the relative sensitiveness of man and woman to physical pain, a comparison by no means easy to estimate on account of the abstract nature of the subject. In the present instance we propose dealing with the differences existing, in the relative sense of smell as exhibited in the two sexes. And here again we must acknowledge our indebtedness to Mr. Havelock Ellis, who has collected together much valuable matter on this question.

The first accurate measurements regarding smell were taken in America by Professor Bailey and Professor Nichols. In an article which he published a year or two back, Mr. Bailey shows us that, with regard to many common things, delicacy of perception is far more marked among men than among women. To show how these conclusions were arrived at, we will give an abstract of the mode of procedure under which our authorities worked, and from which they summarised their results. Firstly, they prepared a series of solutions of (1) oil of cloves, (2) nitrate of amyl, (3) extract of garlic, (4) bromide, (5) cyanide of potassium. Each member of these solutions they describe as being designed half the strength of the preceding one. These were next extended in successive dilutions till it was impossible to detect the substances by their odours. The bottles then were placed about at random to be classified by their sense of smell; a classification requiring a high development of that particular sense.

The first series of experiments which is reported were conducted in the presence of seventeen males and seventeen females, the results being expressed in the following table, which gives the exact amount of each substance detected :—

| | Oil of Cloves. | Nitrite of Amyl. | Garlic. | Bromide | Cyanide. |
|---|---|---|---|---|---|
| Average of Males. | 1 part in 88,218 of water. | 1 in 783,870 | 1 in 57,927 | 1 in 49,254 | 1 in 109,140 |
| Average of Females. | 1 in 50,667 of water. | 1 in 311,330 | 1 in 43,900 | 1 in 16,244 | 1 in 9,002 |

The result of this tabular matter points only in one direction, but the question at once arises in one's mind how much on an equality the persons were who were submitted to these experiments? Were they alike in the experience they had gained in distinguishing substances by smell, for we are all aware that a great facility is obtainable in this direction. But Professor Nichols dismisses the question of inequality at once. The class of individuals he tested were almost entirely students of the University of Kansas, a co-educational institution of fair grade, which at that time contained nearly equal numbers of young men and women, ages from seventeen to twenty-five years, the only distinction which could be drawn, other than sexual, being that which arises from the fact that boys, in an institution offering a considerable choice of studies, select the sciences rather than the letters, gaining thereby some training of the special senses. This we have on Professor Nichols' own authority, in a letter on the subject addressed to Mr. Havelock Ellis. But the few cases in which he deemed it certain that training entered, the American scientist informs us, were those of students of pharmacy who had given much practice in the recognition of drugs, &c., by use of the unaided senses—touch, taste, smell, &c. There was no attempt made, it appears, to select within the class of college students. The experiments were not, indeed, conducted with a view to sexual differences. The male and female were not the same in the various experiments.

The above table of results was not, of course, the only one, nor indeed the one from which our American authorities deduced their conclusions; it was only one of the many which were undertaken. To give another in which twenty-seven males and twenty-one females were engaged points only in the same direction :

| | Prussic Acid. | Oil of Lemon. | Oil of Wintergreen. |
|---|---|---|---|
| Average of Males. | 1 part in 112,000 of water. | 1 part in 280,000 of water. | 1 part in 600,000 of water. |
| Average of Females. | 1 part in 18,000 of water. | 1 part in 116,000 of water. | 1 part in 311,000 of water. |

However, after mature and exhaustive research in this direction, both of the investigators to whom we refer came to the conclusion that the average of their results proved a more delicate sense of smell in man than in woman.

It is interesting to see Professor Nichols' own words on the subject, "It should be said," he writes, "in considering our work, that neither Professor Bailey nor I were experts in the physiology of the senses. His interest in the matter lay in its bearings upon chemistry, mine in its relation to physics. The points of sexual difference met with were not looked for in planning our experiments. They were, almost without exception, just the opposite of our preconceived notions concerning such differences. The number of individuals tested was probably insufficient to enable us to draw very broad conclusions. We deemed the differences worthy of record, however, to be given such weight as their limited character would justify."

Then to turn from American results, we find that Dr. Ottolenghi, in the Laboratory of Forensic Medicine of Turin University, devoted much time and thought to this subject. He made a series of observations on thirty normal men and twenty normal women of the middle and lower classes.* He commenced corresponding nvestigations on eighty criminal men and women. To carry out his scheme, the doctor invented a kind of osmometer with twelve aqueous solutions of essence of clove, ranging in strength from $\frac{1}{5000}$ to $\frac{1}{100}$; in other respects, Mr. Ellis tells us, the methods identically followed on these of Professor Bailey and Nichols. Dr. Ottolenghi selected essence of cloves as being a particularly odorous substance, very fractionable, and well known. His conclusions show that olfactory acuteness was slightly less distinguishable in women than in men.

Our own English authority, in summing up this and much other evidence brought to bear upon the questions, admits that the evidence furnished us internationally, so far as it goes, is uniform in its conclusions, and points to the fact that the sense of smell is possessed in decidedly greater keenness by the male than by the female.

* L'Olfatto nei Criminalé, Archivio di Psichialvia, 1888. Vol. ix., fasc. 5.

